# The effect of integrated health care in patients with hypertension and diabetes: a systematic review and meta-analysis

**DOI:** 10.1186/s12913-022-07838-1

**Published:** 2022-05-05

**Authors:** Yan Zhao, Yue Ma, Chongbo Zhao, Jiahong Lu, Hong Jiang, Yanpei Cao, Yafang Xu

**Affiliations:** 1 Department of Nursing, Huashan Hospital, Fudan University, 12 Middle Urumqi Road, Shanghai, China; 2 Department of Neurology, Huashan Hospital, Fudan University, Shanghai, China; 3 School of Nursing, Fudan University, Shanghai, China

**Keywords:** Integrated health care, Hypertension, Diabetes, Systematic review, Meta-analysis

## Abstract

**Background:**

A growing number of studies show that integrated health care provides comprehensive and continuous care to patients with hypertension or diabetes. However, there is still no consensus about the effect of integrated health care on patients with hypertension or diabetes. The objective of this study was to verify the effectiveness of integrated health care for patients with hypertension or diabetes by using a systematic review and meta-analysis.

**Methods:**

The study searched multiple English and Chinese electronic databases. The search period was from database inception to 31 October 2020. Systematic reviews and meta-analyses were conducted after assessing the risk of bias of each study.

**Results:**

Sixteen studies that involved 5231 patients were included in this study. The results of the systematic review revealed that systolic blood pressure (SBP), diastolic blood pressure (DBP), body mass index (BMI) and glycosylated haemoglobin (HbA1c) are commonly used indicators for patients with hypertension or diabetes. Individual models and group- and disease-specific models are the most commonly used models of integrated health care. All the studies were from high-income and middle-income countries. Meta-analysis showed that integrated health care significantly improved SBP, DBP and HbA1c but not BMI. A comparison of interventions lasting 6 and 12 months for diabetes was conducted, and HbA1c was decreased after 12 months. The changes in SBP and DBP were statistically significant after using group- and disease-specific model but not individual models. HbA1c was significantly improved after using group- and disease-specific models and individual models.

**Conclusion:**

Integrated health care is a useful tool for disease management, and individual models and group- and disease-specific models are the most commonly used models in integrated health care. Group- and disease-specific models are more effective than individual models in the disease management of hypertension patients. The duration of intervention should be considered in the disease management of patients with diabetes, and interventions longer than 12 months are recommended. The income level may affect the model of integrated health care in selecting which disease to intervene, but this point still needs support from more studies.

## Introduction

**Fig. 1:**
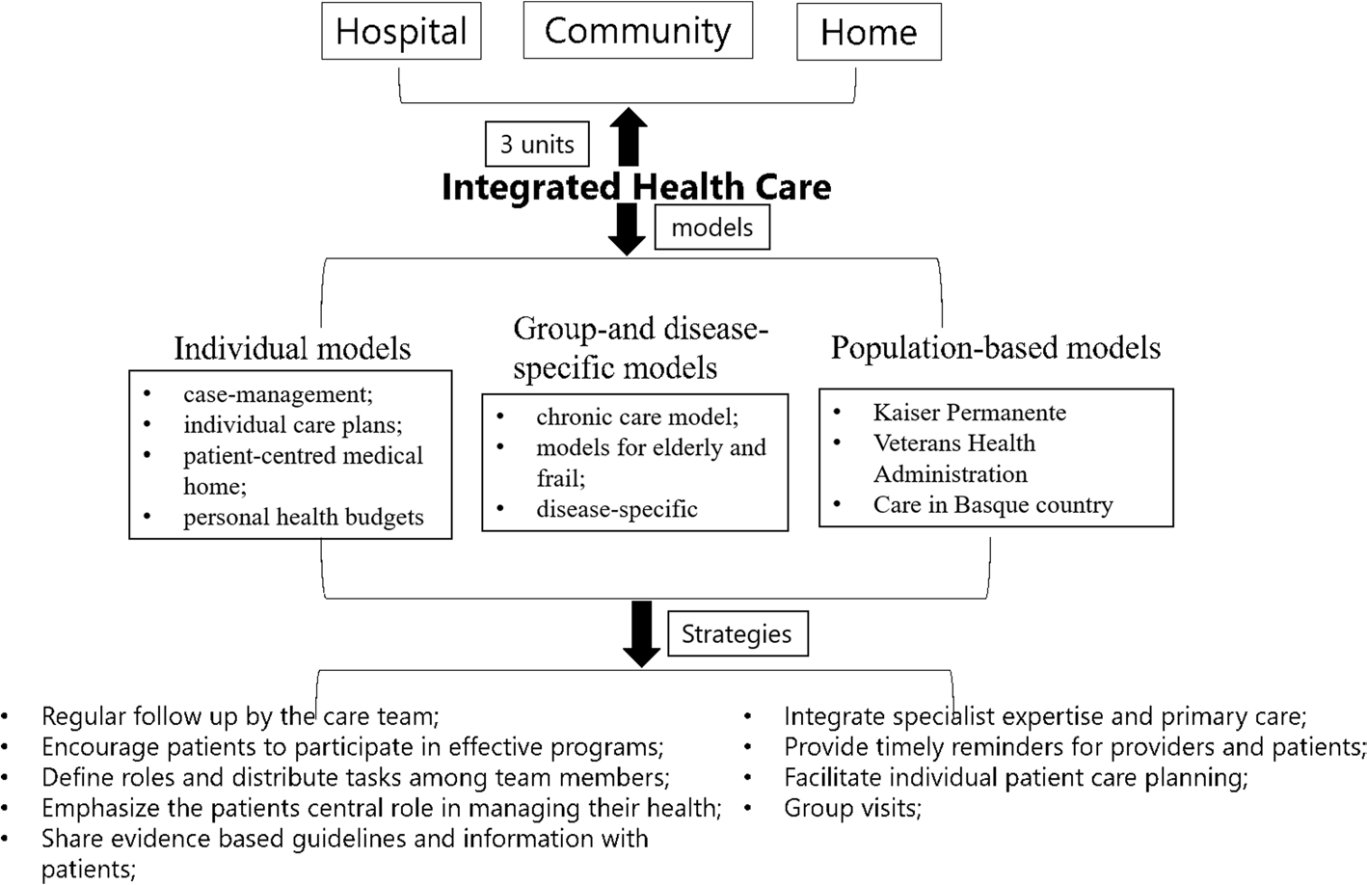
Strategies and methods in integrated health care

Integrated health care is defined as care that requires the coordination of multiple service components and an appropriate infrastructure to supply people with comprehensive and continuous care services [1, 2]. This type of care is widely used for chronic disease management, especially for patients with hypertension or diabetes [3–7]. As the models of integration care reported by WHO, Integrated health care has three types of models [8]: individual models, group- and disease-specific models, and population-based models (Fig. 1). Individual models involve intervention methods such as case management, individual care plans, patient-centred medical care, and personal health budgets. The intervention methods of group- and disease-specific models include chronic care, care for elderly individuals and frail individuals, and disease-specific care. Kaiser Permanente, the Veterans Health Administration, and care in Basque country are population-based models. Population-based models are specific models, in which the participants are widely covered compared with the individual models or group- and disease-specific models. For example, Kaiser Permanente (KP) is the largest integrated care delivery system in the USA. It focuses on chronic care and multispecialty practice and requires membership and prepayment management [9–12]. The Veterans Health Administration provides complementary and integrative health (CIH) services to veterans [13, 14]. Integrated care in the Basque country has emerged with the launch of a strategy to tackle the challenge of chronic diseases in Spain. Basque integrated care strategies are government oriented, so they are still not widely used worldwide [15]. Integrated health care uses various strategies, such as regular follow-up by the care team, encouragement of patients to participate in effective programs, defined roles and distribution of tasks among team members, emphasis on the patients’ central role in managing their health, sharing of evidence-based guidelines and information with patients; integration of specialist expertise and primary care, timely reminders for providers and patients; facilitation of individual patient care planning; group visits; and so on [16].

Chronic diseases have an increasing influence on people’s quality of life due to the long course and high incidence of their disease [17]. Chronic disease can result in permanent organ injury or residual disability caused by nonreversible pathological alterations. Patients may require special education for rehabilitation or are expected to require a long period of supervision, observation, or care. In the American population, 90 % of older adults have at least one chronic condition, with many having two or more [18–21]. Hypertension and diabetes are both common chronic diseases [22]. Hypertension is defined as a systolic blood pressure measurement that is consistently greater than 140 mmHg or a diastolic blood pressure measurement that is consistently at 90 mmHg or higher. The 2017 Global Burden of Disease (GBD) study indicated that hypertension was an important public health challenge worldwide and will affect 1.56 billion individuals by 2025, with an increased global prevalence of 60% [5, 23]. Diabetes is defined as a group of disorders characterized by hyperglycaemia and glucose intolerance. It was responsible for 1.6 million deaths among all deaths from noncommunicable chronic disease in 2016 [24].

The National Health Insurance Service of Korea has emphasized the need to change the healthcare system to one that is centred on the community, as the hospital-centred medical system has brought about many problems, such as medical refugees and care refugees [25]. A growing number of studies have reported models of long-term care for patients with hypertension or diabetes based on integrated health care in the community [26]. However, there is still no consensus about the effectiveness of disease management when integrated health care is used as an intervention. The management of hypertension and diabetes is currently facing the challenge of how to provide healthcare services that integrate hospital-based expertise and community needs [27]. The aim of this study was to verify the effectiveness of integrated health care services from community-hospital for patients with hypertension or diabetes who live in communities with a systematic review and meta-analysis.

## Methods

### Study eligibility

The selected literature met the following inclusion criteria: (1) Eligible participants were those diagnosed with hypertension or diabetes. (2) All the articles should use a type of integrated health care as the intervention method. (3) The study type of the article was a randomized clinical trial. (4) Articles had to be published in Chinese or English.

Articles were excluded if any of the following exclusion criteria were met: (1) the full text could not be found; (2) eligible participants did not meet the inclusion criteria; (3) the intervention did not involve integrated health care; (4) outcome indicators did not meet the study criteria; and (5) any of the data required for the research was not provided.

### Data sources

English databases such as GIN, NICE, Cochrane, JBI, CINAHL, EMBASE, PubMed, Medline, and Web of Science and Chinese databases such as SinoMed, CNKI, WAN FANG and VIP were searched. The searches covered the period beginning with database inception to 31 October 2020.

Search terms were used to retrieve literature from the databases. Example of search strategies in PubMed are shown in Table 1.

**Table 1:** Search strategies in PubMed

	Searches	Results^a^	Type
#1	Search: ((((((((Integrated Health Care[Title/Abstract]) OR (Home Care Services[Title/Abstract])) OR (Hospital-Based Home Care Services[Title/Abstract])) OR (Home Health Nursing[Title/Abstract])) OR (Home Nursing[Title/Abstract])) OR (Comprehensive Health Care[Title/Abstract])) OR (Patient Care Planning[Title/Abstract])) OR (Primary Health Care[Title/Abstract])) OR (Progressive Patient Care[Title/Abstract])	34,350	ADVANCED
#2	Search: (((((((((case management[Title/Abstract]) OR (individual care plans[Title/Abstract])) OR (patient-centred medical home[Title/Abstract])) OR (personal health budgets[Title/Abstract])) OR (chronic care model[Title/Abstract])) OR (models for elderly[Title/Abstract] AND frail[Title/Abstract])) OR (disease-specific[Title/Abstract])) OR (Kaiser Permanente[Title/Abstract])) OR (Veterans Health Administration[Title/Abstract])) OR (care in Basque country[Title/Abstract])	49,857	ADVANCED
#3	Search: ((Hypertension[Title/Abstract]) OR (Diabetes[Title/Abstract])) OR (Diabetes Mellitus[Title/Abstract])	866,706	ADVANCED
#3	#1 AND #2 AND #3	91	ADVANCED

^a^ Deadline to 31 October 2020.

### Study selection

We searched articles based on the search terms. Articles were excluded if, after reading the title, abstract and full text, they met the exclusion criteria. The remaining articles were included in the meta-analysis.

### Data extraction

Two investigators extracted data from each study independently. A data extraction sheet was used to extract the following data: authors, journal, date, country, study type, participants, experiment and control sample size, disease, intervention methods, outcome index, experiment duration and main conclusion.

### Risk of bias assessment

The quality of each study was evaluated by the risk-of-bias assessment tool recommended by the Cochrane Handbook for Systematic Reviews of Interventions-version 5.1.0 [28].

Meta-analysis was performed with RevMan5.4 software [29]. Clinical heterogeneity of each study was evaluated based on the inclusion and exclusion criteria. The heterogeneity of the study design was assessed according to the content and completeness of the outcome indicators, duration of intervention, and randomization methods.

### Data synthesis

Meta-analysis was performed on the organized and summarized outcome measurements from at least three articles. Since all outcomes (e.g., systolic blood pressure, diastolic blood pressure, body mass index and glycosylated haemoglobin) were continuous data, they were analysed with the weighted mean difference. The 95% confidence interval (CI) was also calculated, and the meta-analysis test level was set at *p* = 0.05. I^2^ statistics were used to assess heterogeneity of effect size, and I^2^ statistic was used to check for inconsistencies between the studies (I^2^ = 0–100%; greater than 50% was considered significant statistical heterogeneity). A fixed-effect model was applied if the heterogeneity from multiple studies was small (*p* > 0.05 with I^2^ ≤ 50%). Otherwise, a random-effects model was adopted if there was high heterogeneity (*p* < 0.05, I^2^ ≥ 50%). Potential publication bias was assessed by a funnel plot.

## Results

### Study selection and characteristics

**Fig. 2:**
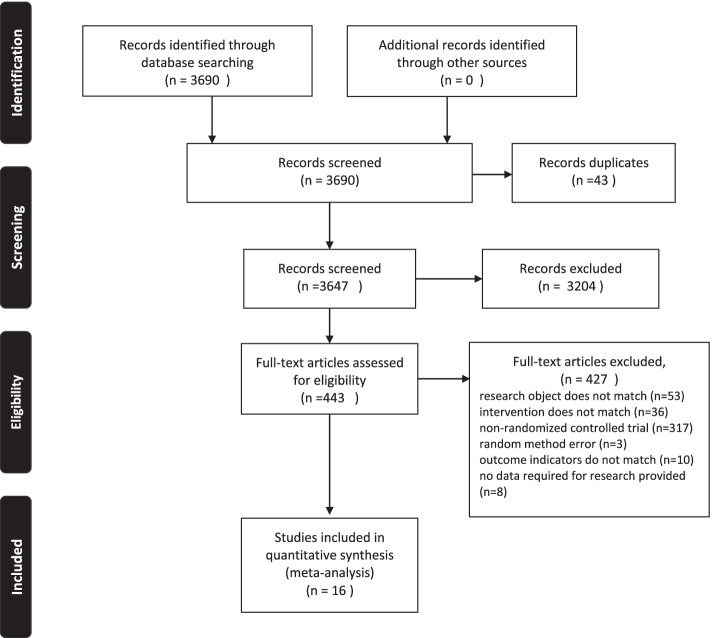
Flow of studies throughout the review

The details of the selection process are shown in Fig. 2. Sixteen articles [4–7, 30–41] were included in this study. The publication years of the studies ranged from 1993 to 2020. Five articles reported data from China; four, America; one, Brazil; one, Finland; one, the UK; one, Turkey; one, Italy; one, the Netherlands; and one, Spain.

**Fig. 3:**
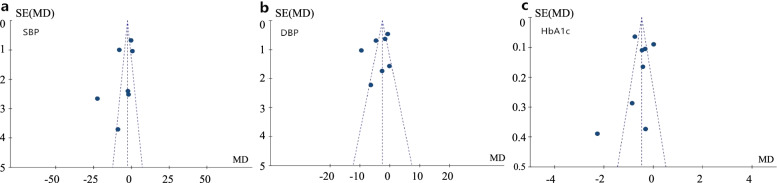
Funnel plot for publication bias. **a** Funnel plot for SBP in the patients with hypertension. **b** Funnel plot for DBP in the patients with hypertension. **c** Funnel plot for HbA1c in the patients with diabetes. *SE: standard error; MD: mean difference; SBP: systolic blood pressure; DBP: diastolic blood pressure; HbA1c: glycated haemoglobin

Sixteen articles (seven on hypertension and nine on diabetes) involved 5231 patients with diabetes or hypertension who lived in communities (2593 with diabetes and 2638 with hypertension). The characteristics of the studies are described in Table 2. The summary of model are also described in Table 3. The funnel plots for blood pressure, including systolic blood pressure, diastolic blood pressure and glycosylated haemoglobin, are shown in Fig. 3, and no publication bias was found.

**Table 2:** Characteristics of included randomized controlled trial studies (*n* = 16)

Authors, years	Participants	Intervention	Intervention providers	Outcome measures	Duration	Conclusion
Mattei da Silva, ÂT et al. 2020 [7], Brazil	94 patients with hypertension Experiment group: 1) Age: 49.4 ± 6.4 2) Size: 47 Control group: 1) Age: 49.2 ± 8.4 2) Size: 47	**Case management:** Evidence-based guidelines and information; Timely reminder; Regular follow up; Individual care planning	Nurses	•SBP*, DBP^*^ •BMI^*^	12 months	The intervention group’s SBP*, DBP* and BMI* decreased significantly compared to those of the control group
Kastarinen MJ et al. 2002 [36], Finland	587 patients with hypertension Experiment-group: 1) Age: 54.4 ± 10.1 2) Size: 304 Control-group: 1) Age: 54.2 ± 9.9 2) Size: 283	**Disease-specific:** Evidence-based guidelines and information; Regular follow up; Patients participate in effective programs	Physician and a nutritionist; Local public health nurses	•SBP*, DBP^*^ •BMI^*^	12 months	Significant reductions after 1 year both in SBP* and in DBP* were in favour of the intervention group.
Aubert RE et al. 2017 [30], America	138 patients with diabetes Experiment-group: 1) Age: 53 (Interquartile range: 47–61) 2) Size: 71 Control-group: 1) Age: 54 (Interquartile range: 46–60) 2) Size: 67	**Case management:** Evidence-based guidelines and information; Timely reminder; Regular follow up; Individual care planning; Patients participate in effective programs	a board-certified family medicine physician and an endocrinologist; Local public health nurses	•HbA1c*	12 months	A significant decrease in HbA1c* in intervention group.
Hurwitz B et al. 1993 [35], British	181 patients with diabetes Experiment-group: 1) Age: 62.0 ± 11.2 2) Size: 89 Control-group: 1) Age: 63.1 ± 8.6 2) Size: 92	**Patient-centred medical home:** Evidence-based guidelines and information; Regular follow up; Timely reminder	General practitioners; Clinic doctors	•HbA1c*	6 months	Decrease in HbA1c* in intervention group.
Gao JL et al. 2015 [6], China	1204 patients with hypertension Experiment-group: 1) Age: 66.0 ± 9.3 2) Size: 600 Control-group: 1) Age: 67.1 ± 10.3 2) Size: 604	**Patient-centred medical home:** Evidence-based guidelines and information; Regular follow up; Individual care planning; Patients participate in effective programs	General practitioners	•SBP*, DBP* •BMI*	12 months	The average DBP* decrease in the intervention group was significantly greater than that in the control group.
Hacihasanoğlu R et al. 2010 [5], Turkey	80 patients with hypertension Experiment-group: 1) Age: 56.92 ± 8.04 2) Size: 40 Control-group: 1) Age: 55.62 ± 8.46 2) Size: 40	**Patient-centred medical home:** Evidence-based guidelines and information; Timely reminder; Regular follow up; Individual care planning; Patients participate in effective programs	Nurses	•SBP*, DBP* •BMI*	12 months	A significant decrease in BP* in intervention group.
Li YD et al. 2003 [40], China	415 patients with diabetes Experiment-group: 1) Age: 66.77 ± 7.29 2) Size: 215 Control-group: 1) Age: 67.95 ± 7.61 2) Size: 200	**Disease-specific:** Evidence-based guidelines and information; Regular follow up; Timely reminder; Patients participate in effective programs; Group visit	staff in general hospital; staff in community hospital	•HbA1c*	12 months	A significant decrease in BP* and HbA1c* in the intervention group.
Huang HL et al. 2019 [34], China	222 patients with diabetes Experiment-group: 1) Age: 68.3 ± 5.1 2) Size: 110 Control-group: 1) Age: 68.2 ± 5.4 2) Size: 112	**Disease-specific:** Evidence-based guidelines and information; Regular follow up; Individual care planning; Patients participate in effective programs	specialist physician general practitioner	•HbA1c*	6 months 12 months	A significant decrease in BP* in the intervention group after 3, 6, and 12 months. A significant decrease in HbA1c* in intervention group after 6 months.
Han Y et al. 2019 [41], China	100 patients with diabetes Experiment-group: 50 Control-group: 50 Age: 25 ~ 75	**Disease-specific:** Evidence-based guidelines and information; Regular follow up; Individual care planning	staff in general hospital; staff in community hospital	•HbA1c*	6 months 12 months	A significant decrease in HbA1c* in the intervention group after 12 months.
Gary TL et al. 2009 [33], America	488 patients with diabetes Experiment-group: 1) Age: 59 ± 11 2) Size: 235 Control-group: 1) Age: 56 ± 11 2) Size: 253	**Case management:** Evidence-based guidelines and information; Timely reminder; Regular follow up; Individual care planning; Patients participate in effective programs	Nurses; Community health worker	•HbA1c*	24 months	Those who had more visits with professional workers in intervention group had a statistically significant decline in HbA1c* level compared with the control group.
Glasgow RE et al. 2005 [39], America	733 patients with diabetes Experiment-group: 1) Age: 62 ± 1.4 2) Size: 379 Control-group: 1) Age: 64 ± 1.3 2) Size: 354	**Disease-specific:** Evidence-based guidelines and information; Timely reminder; Regular follow up; Individual care planning; Patients participate in effective programs	Primary care physician; care manager	•HbA1c*	12 months	Both conditions improved on measures of HbA1c*, but there was not a significant difference between conditions.
Piatt GA et al. 2006 [4], America	73 patients with diabetes Experiment-group: 1) Age: 69.7 ± 10.7 2) Size: 27 Control-group: 1) Age: 68.6 ± 8.6 2) Size: 46	**Chronic Care Model:** Evidence-based guidelines and information; Timely reminder; Regular follow up; Individual care planning; Patients participate in effective programs	Physician; Nurses practitioners/physician assistants Behaviourist	•HbA1c*	12 months	A marked decline in HbA1c* was observed in the intervention group but not in the control group.
Kong JXx et al. 2018 [37], China	258 patients with diabetes Experiment-group: 1) Age: 69.12 ± 10.54 2) Size: 134 Control-group: 1) Age: 71.48 ± 8.79 2) Size: 124	**Chronic Care Model:** Evidence-based guidelines and information; Timely reminder; Regular follow up; Individual care planning; Patients participate in effective programs	Physicians	•HbA1c*	9 months	The intervention group had a remarkable reduction in HbA1c*.
Cicolini G et al. 2014 [32] Italy	298 patients with hypertension Experiment-group: 1) Age: 59.8 ± 15.0 2) Size: 100 Control-group: 1) Age: 58.3 ± 13.9 2) Size: 98	**Disease-specific:** Evidence-based guidelines and information; Regular follow up; Patients participate in effective programs	Nurses	•SBP*, DBP* •BMI*	6 months	The intervention group showed a significantly greater improvement in BMI* and SBP* and DBP*.
Beune EJ et al. 2014 [31], Netherlands	139 patients with hypertension Experiment-group: 1) Age: 53.3 ± 10.2 2) Size: 71 Control-group: 1) Age: 54.6 ± 9.5 2) Size: 68	**Disease-specific:** Individual care planning; Regular follow up; Patients participate in effective programs	Nurses	•SBP*, DBP* •BMI*	6 months	In contrast to SBP* and BMI*, effect of the intervention on the between-group difference in DBP* reduction was significant.
Leiva A et al. 2014 [38], Spain	208 patients with hypertension Experiment-group: 1) Age: 64.5 ± 9.8 2) Size: 103 Control-group: 1) Age: 66.7 ± 11.7 2) Size: 105	**Disease-specific:** Evidence-based guidelines and information; Timely reminder; Regular follow up; Family support; Patients participate in effective programs	Nurse	•SBP*, DBP*	12 months	The SBP* in the intervention group was 151.3 versus 153.7 in the control group (*P* = 0.294). The DBP* did not differ between groups (83.4 versus 83.6).

*BP Blood pressure, *SBP* Systolic blood pressure, *DBP* Diastolic blood pressure, *BMI* Body Mass Index, *HbA1c* Glycated haemoglobin.

**Table 3:** Summary of the models

Type of integration	Disease	Author	Model
Individual Model	Diabetes	Aubert RE et al.(2017) [30]	Case management
		Hurwitz B et al.(1993) [35]	Patient-centred medical home
		Gary TL et al.(2009) [33]	Case management
	Hypertension	Mattei da Silva, ÂT et al.(2020) [7]	Case management
		Gao JL et al. (2015) [6]	Patient-centred medical home
		Hacihasanogu R et al.(2010) [5]	Patient-centred medical home
Group-and disease-specific model	Diabetes	Li YD et al.(2003) [40]	Disease-specific
		Huang HL et al.(2019) [34]	Disease-specific
		Han Y et al.(2019) [41]	Disease-specific
		Glasgow RE et al.(2005) [39]	Disease-specific
		Piatt GA et al.(2006) [4]	Chronic Care Model
		Kong JX et al.(2018) [37]	Chronic Care Model
	Hypertension	Kastarinen MJ et al.(2002) [36]	Disease-specific
		Cicolini G et al. (2014) [32]	Disease-specific
		Beune EJ et al. (2014) [31]	Disease-specific
		Leiva A et al. (2014) [38]	Disease-specific

### Methodological quality

**Fig. 4:**
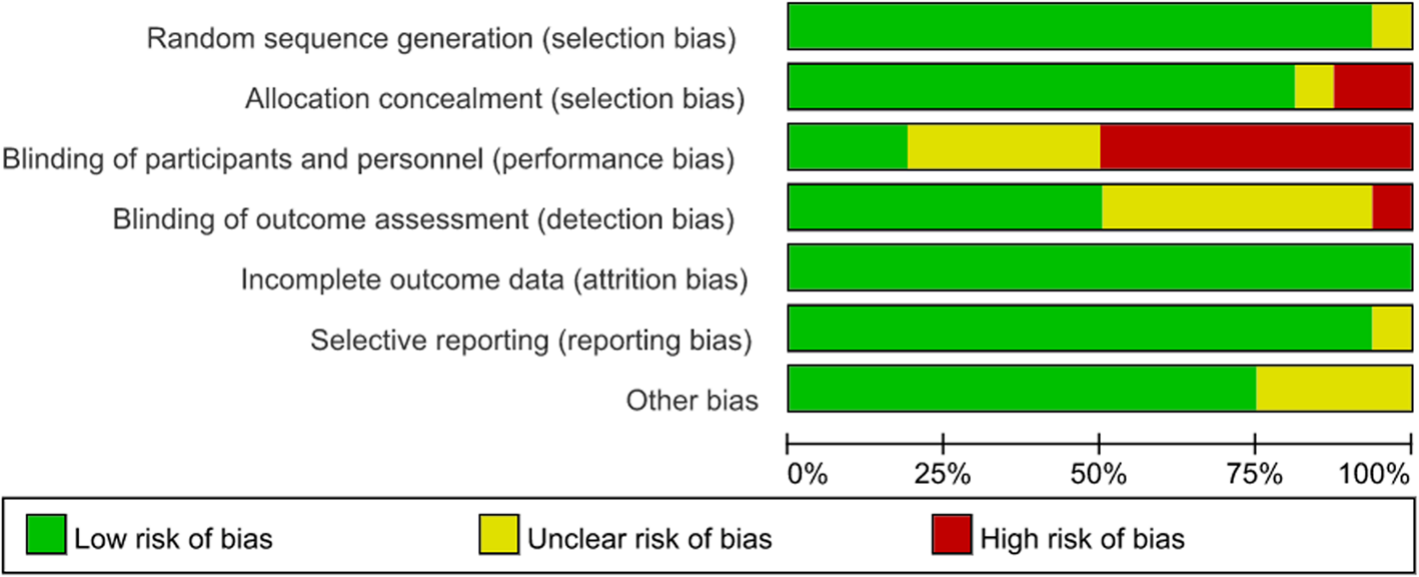
Summaries of bias. * green = low risk of bias, red = high risk of bias, yellow = unclear risk of bias

Sixteen articles were included in the risk-of-bias assessment (Fig. 4). Fifteen of the articles [4, 5, 7, 30–41] used the random group design, one article [6] did not elaborate on the randomization methods used. Thirteen studies [4, 5, 7, 30–36, 38, 39, 41] used effective methods to reduce the risk of allocation bias, such as the use of opaque envelopes and undisclosed random number tables. Eight articles [4, 5, 7, 31, 33, 37, 38, 41] had a high risk related to performance. Eight articles [5–7, 31–33, 37, 38] used blinding of outcome assessment. All of the included articles reported lost data and subjects that were lost to follow-up.

### Systematic review

#### Common outcome measures of integrated health care used in the management of patients with hypertension or diabetes

Seven articles [5–7, 31, 32, 36, 38] focused on hypertension, and the values of systolic blood pressure (SBP) and diastolic blood pressure (DBP) were used in these articles. Six articles reported body mass index (BMI). Blood pressure was a major risk factor for heart disease in the population [42], and SBP and DBP were measured as the primary outcomes to assess blood pressure in the study. BMI is a classic index used to assess the potential risk associated with hypertension [43–46]. Nine articles [4, 30, 33–35, 37, 39–41] used glycosylated haemoglobin (HbA1c) as their primary outcome in patients with diabetes. HbA1c is formed by a progressive, nonenzymatic reaction between glucose and haemoglobin in erythrocytes [47]. It is a measure of average blood glucose over several months, so it is the most common index used to assess the severity of diabetes [48]. The advantage of this test for screening in the community is that it can be checked in a nonfasting, random fashion [49]. Fasting blood glucose (FBG) was also reported in two articles, but there were not sufficient data to perform the meta-analysis [4, 39].

#### Common models of integrated health care used in the management of patients with hypertension or diabetes

Individual models and group- and disease-specific models are common models of integrated health care for use with patients with hypertension or diabetes. Hacihasanoğu et al. and Mattei et al. used individual models of integrated health care to perform disease management for patients with hypertension for 12 months. The results of the two studies indicated that there was a significant difference in SBP and DBP between the experimental group and the control group and that an individual model could improve the blood pressure of hypertension patients [5, 7]. Gao et al. also used an individual model as an intervention to follow up hypertension patients for 6 months. The results showed that there was a significant difference in DBP between the intervention and control groups but not in SBP [6]. Beune et al. and Cicolini et al. used group- and disease-specific models to manage hypertension patients for 6 months, and the results indicated that a group- and disease-specific model could reduce SBP, DBP and BMI [31, 32]. The results of Kastarinen et al. showed that group- and disease-specific models could be used to effectively manage SBP and DBP in the proper range after 12 months of intervention, but it could not be used to reduce BMI [36].

Individual models were also used in the diabetes studies by Aubert et al. and Gary et al. for 12 months and 24 months. All of the results indicated a statistically significant improvement in HbA1c in the intervention group [30, 33]. Hurwitz et al. also used an individual model as the intervention for integrated health care for 6 months, but they obtained the opposite result: the model could not be used to improve HbA1c [39].

#### Intervention duration of integrated health care used in the management of patients with hypertension or diabetes

We found that intervention duration was an important index that had an extensive impact on outcomes of patients with diabetes. Three articles reported the outcome after intervention for 6 months, and 7 articles reported the outcome after intervention for 12 months. Huang et al. and Han et al. used group- and disease-specific models as interventions [34, 41]. Huang et al. found that the difference between the intervention group and control group was statistically significant after 6 months rather than 3 months [34]. Han et al. assessed an intervention for 3, 6 and 12 months and found that HbA1c began to decrease when the intervention had been performed for 12 months [41].

#### Integrated health care intervention between high- and middle-income countries

All of the studies were from high-income and middle-income countries: seven articles were from middle-income countries [5–7, 34, 37, 40, 41], and nine articles were from high-income countries [4, 30–33, 35, 36, 38, 39]. Among the articles from middle-income countries, three studies [5–7] used individual models as interventions in patients with hypertension, and four studies [34, 37, 40, 41] used group- and disease-specific models as interventions in patients with diabetes. Among the articles from middle-income countries, three articles [30, 33, 35] used individual models as interventions in patients with diabetes, four articles [31, 32, 36, 38] used group- and disease-specific models for hypertension patients, and two articles [4, 39] used group- and disease-specific models in diabetes patients.

#### Meta-analysis of integrated health care in patients with hypertension

Seven articles [5–7, 31, 32, 36, 38] reported SBP and DBP, and 6 articles [5–7, 31, 32, 36] reported BMI in patients with hypertension. Meta-analyses were performed on SBP, DBP, and BMI.

**Fig. 5:**
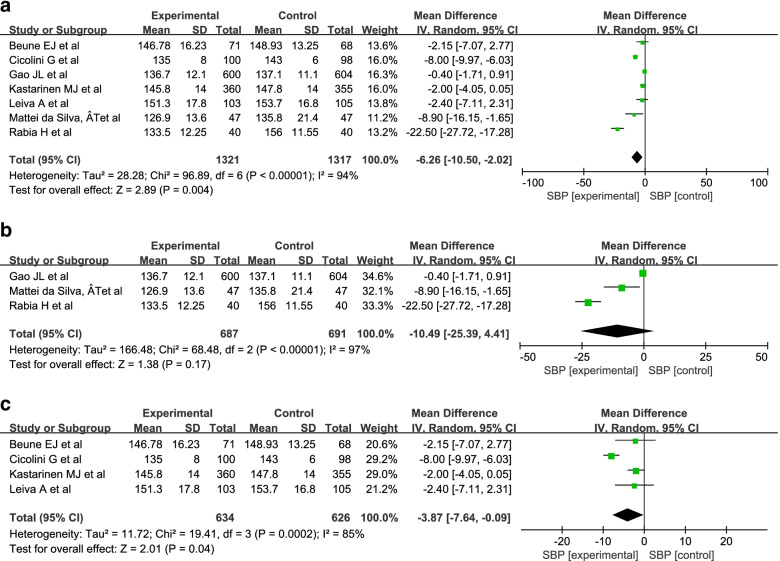
Meta-analysis of SBP in patients with hypertension. **a** The meta-analysis of SBP in patients with hypertension. **b** The meta-analysis of SBP in patients involved in individual model. **c** The meta-analysis of SBP in patients involved in Group-and disease-specific model

The results showed that I^2^ value were 94% (Fig. 5a); 97% (Fig. 5b); 85% (Fig. 5c), they indicated high heterogeneity, so we adopted the random-effect model. The results of the meta-analysis showed that there was a significant difference in SBP between the intervention group and the control group (MD = -6.26; 95% Cl = − 10.50, − 2.02; *P* = 0.004) (Fig. 5a). Because individual models and group- and disease-specific group models are commonly used, we conducted a meta-analysis of these two groups. The results showed a significant difference between the intervention group and the control group in group- and disease-specific models (MD = -3.87; 95% Cl = − 7.64, − 0.09; *P* = 0.04) but not in individual models (MD = -10.49; 95% Cl = − 25.39, 4.41; *P* = 0.17). (Fig. 5b, c).

**Fig. 6:**
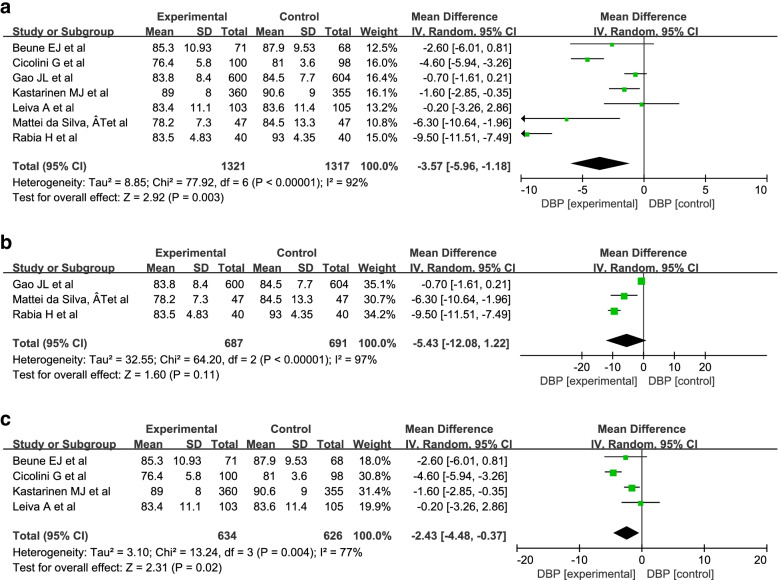
Meta-analysis of DBP in patients with hypertension. **a** The meta-analysis of DBP in patients with hypertension. **b** The meta-analysis of DBP in patients involved in individual model. **c** The meta-analysis of DBP in patients involved in Group-and disease-specific model

A random-effect model was used into the analysis of DBP since there was high statistical heterogeneity (I^2^ = 92%; 97%; 77%). The results of the meta-analysis showed that there was a significant difference in DBP between the intervention group and the control group (MD = -3.57; 95% Cl = − 5.96, − 1.18; *P* = 0.003) (Fig. 6a). Because individual models and group- and disease-specific group models are commonly used, we conducted a meta-analysis of these two groups. The results showed that there were significant differences between the intervention group and the control group in the group- and disease-specific models (MD = -2.43; 95% Cl = − 4.48, − 0.37; *P* = 0.02) but not in the individual models (MD = -5.43; 95% Cl = − 12.08, 1.22; *P* = 0.11). Figure 6b and c show that the result was same as that of SBP.

**Fig. 7:**
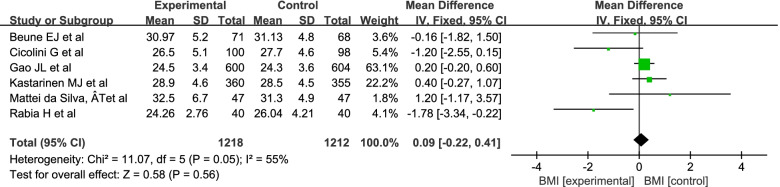
Meta-analysis of BMI in patients with hypertension

A random-effects model was used into the analysis of BMI since there was high statistical heterogeneity(I^2^ = 55%). The results of the meta-analysis showed that there was no significant difference between the intervention group and the control group (MD = -0.14; 95% Cl = − 0.78, 0.49; *P* = 0.66) (Fig. 7).

#### Meta-analysis of integrated health care in patients with diabetes

Nine articles [4, 30, 33–35, 37, 39–41] reported HbA1c in patients with diabetes. Meta-analyses were performed on HbA1c and duration of intervention.

**Fig. 8:**
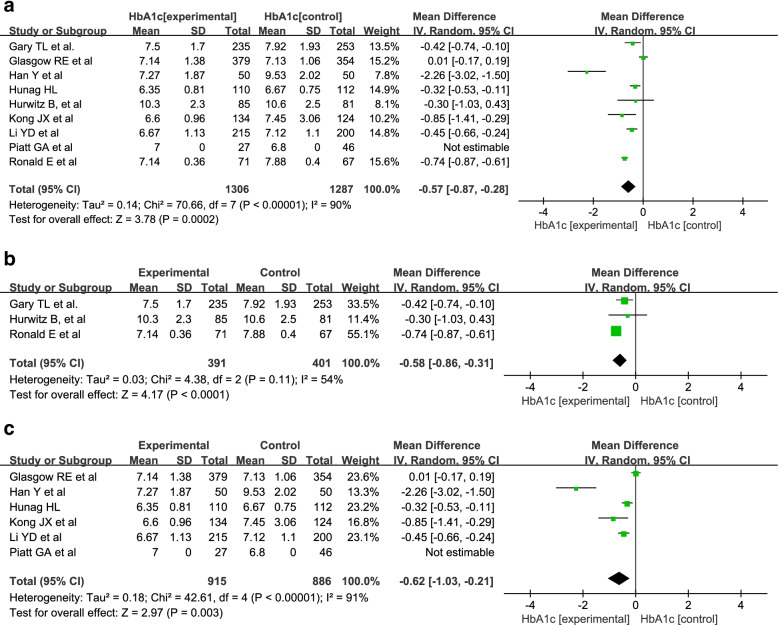
Meta-analysis of HbA1c in patients with diabetes. **a** The meta-analysis of HbA1c in patients with hypertension. **b** The meta-analysis of HbA1c in patients involved in individual model. **c** The meta-analysis of HbA1c in patients involved in Group-and disease-specific model

A random-effects model was used into the analysis of HbA1c since there was high statistical heterogeneity (I^2^ = 90, 54, 91%). The results of the meta-analysis showed that there was a significant difference in HbA1c between the intervention group and the control group (MD = -0.57; 95% Cl = − 0.87, − 0.28; *P* = 0.0002) (Fig. 8a). We also conducted a meta-analysis of individual models and group- and disease-specific models. The results showed that there were significant differences between the intervention group and the control group in both individual models (MD = -0.58; 95% Cl = − 0.86, − 0.31; P<0.0001) and group- and disease-specific models (MD = -0.62; 95% Cl = − 1.03, − 0.21; *P* = 0.003). (Fig. 8b, c).

**Fig. 9:**
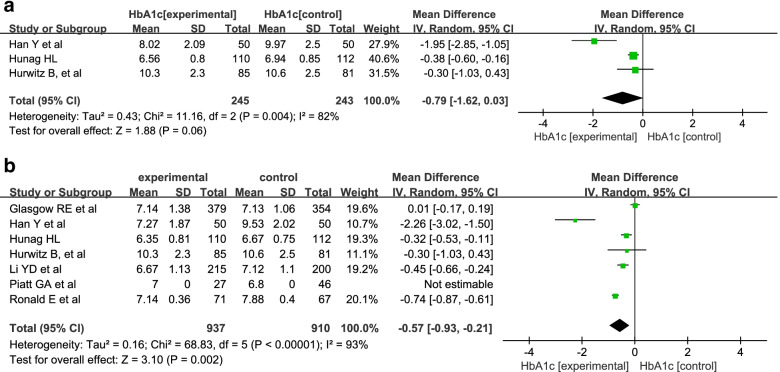
The meta-analysis of HbA1c after 6 and 12 months of intervention. **a** The meta-analysis of HbA1c after 6-months intervention. **b** The meta-analysis of HbA1c after 6-months intervention

A random-effects model was used into the analysis of HbA1c since there was high statistical heterogeneity (I^2^ = 82%; 93%). A meta-analysis of different durations was also conducted. The results revealed that HbA1c was improved after 12 months of intervention (MD = -0.57; 95% Cl = − 0.93, − 0.21; *P* = 0.002) but not after 6 months (MD = -0.79; 95% Cl = − 1.62, 0.03; *P* = 0.08). (Fig. 9).

## Discussion

### Individual models and group- and disease-specific models are the most commonly used models in integrated health care

The study indicated that the individual models and group- and disease-specific models were the most commonly used models in integrated health care. Individual models aim to facilitate the appropriate delivery of health care services and overcome fragmentation between providers [8]. They include case management, individual care plans, patient-centred medical homes, and personal health budgets. Group- and disease-specific models could benefit to encourage better outcomes and quality through leadership and support, it also could mobilize and coordinate resources to patients and motivated to health care team [8]. Regardless of individual models or group- and disease-specific models, the patients these models serve is more universal than that of population-based models, and they have strong operability. Additionally, population-based models always have national characteristics (e.g., Kaiser Permanente and the Veterans Health Administration are unique to United States and care in Basque country is unique to Spain). Therefore, this may be the reason why individual models and group- and disease-specific models are preferred in integrated health care.

### Integrated health care has a positive effect in patients with hypertension

Our study showed that integrated health care has a positive effect on SBP and DBP in patients with hypertension. The results of the meta-analysis also indicated that group- and disease-specific models could improve hypertension and diabetes compared with individual models. Patients with chronic diseases are one of the target groups involved in group- and disease-specific models. Hypertension is one of the four major chronic diseases worldwide. Historical models of clinical care have been developed for acute illness management and are less able to meet the complicated needs of the increasing burden of chronic care [50]. Among the individual model in hypertension, patient-centred medical home and case management were frequently used. Among the group-and disease-specific model in hypertension, disease-specific model was commonly used. The model could involve available community resources and local governmental policies to create a chronic disease care-friendly environment, and they help patients acquire self-management skills and clinical resources [51]. Therefore, group- and disease-specific models of integrated health care are considered more suitable for blood pressure management of patients with hypertension.

We also conducted a meta-analysis on BMI in patients with hypertension, and the results showed that there was not a significant difference in BMI. Of the included studies, we found that few studies recorded and analysed dietary intake and physical activity as observed indicators. Hacihasanoğlu et al. pointed out that guiding patients to reduce calorie intake and increase physical activity can help them control weight [5]. Therefore, future studies can analyse the condition of dietary intake and physical activity in hypertensive patients.

### Integrated health care has a positive effect in patients with diabetes

Our study showed that individual models and group- and disease-specific models of integrated health care may have positive effects on HbA1c in patients with diabetes. Among the individual model in diabetes, case management and patient-centred medical home were adopted. Among the group-and disease-specific model in diabetes, disease-specific model and chronic care model were adopted in the studies included in our research. The management of diabetic patients includes three aspects: diet control, exercise management and drug intervention [52]. Group- and disease-specific models can provide a good chronic disease management environment for patients with diabetes [53], while individual models may provide more accurate health services and long-term follow-up for patients with diabetes. Individual models may help to improve the therapeutic compliance of patients with diabetes, but it still needs more study to verify the opinion in future.

The results of the meta-analysis indicated that after using the integrated health care intervention method for 12 months, the patient’s HbA1c was significantly improved compared to that after a 6-month intervention. This result is similar to the conclusion of Huang et al. [34]. Their study revealed that HbA1c began to decrease when the intervention had been performed for 12 months. Diabetes management is a long-term process, and as the intervention period increases, HbA1c may be improved better.

### Different income-level countries use different levels of intervention with integrated health care in the management of hypertension or diabetes

There are differing socioeconomic, cultural, geographical, political, and health systems in different countries [3]. Middle-income countries are in the primary stage of health service reform. They are facing problems of imperfect care pathways and guidelines, a low standard of state education, and weak health care regulatory mechanisms [54, 55]. High-income countries still face the problems of significant inequities in health services and high costs for complex care needs [56, 57]. These conditions provided the context in which integrated health care was adopted [3]. We have summarized the integrated health care interventions in high- and middle-income countries in our study. The results showed that middle-income countries used individual models to manage patients with hypertension and group- and disease-specific models to manage patients with diabetes. In contrast to middle-income countries, high-income countries used individual models to manage patients with diabetes and group- and disease-specific models to manage patients with hypertension. The income level of the country may affect the model of integrated health care selected for the intervention of different diseases.

### Strengths and potential limitations

In this study, the effectiveness of integrated health care in patients with hypertension or diabetes was studied. Our results indicated that integrated health care can effectively reduce the HbA1c of diabetic patients and that it had a significant effect on the reduction in blood pressure in patients with hypertension compared to patients who received usual care. Thus, hypertension or diabetes management based on integrated health care should be used as a management method for patients with hypertension or diabetes.

Some limitations should be pointed out. First, we did not conduct a meta-analysis on the effect of the intervention duration of integrated health care in patients with hypertension due to a lack of sufficient data in the articles included in our study. Second, this study analysed the indicators of HbA1c, BP and BMI, which focus on diagnosis and treatment. In future studies, we should include more articles that report the intervention duration in hypertension patients, and the index of improvement for patient quality of life should be analysed.

## Conclusion

In this study, a systematic review and meta-analysis was performed to verify the effectiveness of integrated health care in patients with hypertension or diabetes. Integrated health care is a useful tool for disease management, and individual models and group- and disease-specific models are the most commonly used models in integrated health care. Group- and disease-specific models are more effective than individual models in disease management for hypertension. The duration of intervention should be considered in the disease management of patients with diabetes, and interventions longer than 12 months are recommended. The income level of a country may affect the model of integrated health care selected for intervention of different diseases, but this point still needs support from more research.

## Data Availability

All data generated or analysed during this study are included in this published article.
